# Association Between Sleep Disorders and Breast Cancer: An Analysis Based on NHANES and Mendelian Randomization

**DOI:** 10.14740/wjon2825

**Published:** 2026-09-04

**Authors:** Si Yan Chen, Jian Di Li, Ke Jun Wu, Jia Ming Zhang, Liu Mei Zhou, Guo Qiang Chen, Bei Bei Huang, Jing Wen Ling, Gang Chen, Jian Dai

**Affiliations:** a Day Chemotherapy Center, The First Affiliated Hospital of Guangxi Medical University, Nanning 530021, Guangxi Zhuang Autonomous Region, China; b Department of Pathology, The First Affiliated Hospital of Guangxi Medical University, Nanning 530021, Guangxi Zhuang Autonomous Region, China; c Foreign Languages School, Guangxi Medical University, Nanning 530021, Guangxi Zhuang Autonomous Region, China; d Department of Medical Information Engineering, School of Information and Management, Guangxi Medical University, Nanning 530199, Guangxi Zhuang Autonomous Region, China; e Department of Clinical Psychology, Jiangbin Hospital of Guangxi Zhuang Autonomous Region, Nanning 530021, Guangxi Zhuang Autonomous Region, China; f These authors contributed equally to this work.

**Keywords:** Mendelian randomization, National Health and Nutrition Examination Survey, Sleep disorders, Breast cancer

## Abstract

**Background:**

This study aimed to assess the association between sleep disorders and breast cancer (BC) in the National Health and Nutrition Examination Survey (NHANES) and to evaluate potential causal associations using Mendelian randomization (MR), with separate analyses of estrogen receptor-positive (ER+) and estrogen receptor-negative (ER−) BC.

**Methods:**

This study was based on cross-sectional data from the NHANES 2007–2018, including a total of 8,366 female participants. A weighted multivariable logistic regression model was used to analyze the association between sleep disorders and BC, with stepwise adjustment of covariates. Further subgroup analyses were conducted by covariates. Two-sample MR was applied to evaluate the potential causal association between sleep disorders and BC (including ER+ and ER− subtypes).

**Results:**

Analysis of NHANES 2007–2018 cross-sectional data showed that among 8,366 female participants, 33.8% of BC patients reported sleep disorders, which was significantly higher than that in the non-cancer group (27.9%, P = 0.035). However, in the weighted logistic regression model with multivariable adjustment, no significant association was observed between sleep disorders and BC risk (model 3: odds ratio (OR) = 0.814, 95% confidence interval (CI): 0.553–1.196, P = 0.298). Subgroup analysis revealed a significant positive correlation between sleep disorders and BC risk in Mexican Americans (OR = 2.801, 95% CI: 1.302–6.025) and participants with low body mass index (BMI) (OR = 2.705, 95% CI: 1.332–5.494), whereas a significant negative correlation was found in diabetic patients (OR = 0.383, 95% CI: 0.199–0.738). Two-sample MR showed no significant causal effect of sleep disorders on ER+ BC risk (inverse variance weighted (IVW) method: OR = 0.98, 95% CI: 0.94–1.02, P = 0.304). For ER− BC, IVW showed a modest positive association (OR = 1.070, 95% CI 1.013–1.130; P = 0.016), whereas the other MR methods were not statistically significant. No significant bias was detected by heterogeneity test (Cochran’s Q test, P > 0.05) or pleiotropy analysis (MR-Egger intercept test, P = 0.662). Leave-one-out sensitivity analysis and funnel plot further confirmed the robustness and symmetry of the results.

**Conclusion:**

The cross-sectional analysis found no overall association between sleep disorders and BC. MR provided suggestive evidence of a modest association between genetically predicted sleep disorders and ER−, but not ER+, BC; this finding requires cautious interpretation and further validation.

## Introduction

Breast cancer (BC) is one of the most common malignant tumors among women worldwide, with its incidence and mortality consistently on the rise, posing a serious threat to women’s health [1, 2]. In recent years, beyond traditional risk factors, sleep disorders—recognized as a potential modifiable factor—have increasingly garnered the attention of researchers [3]. Numerous studies have demonstrated that the prevalence of sleep disorders in BC patients is significantly higher than in the general population, and it is closely associated with treatment efficacy, quality of life, and survival time [4, 5]. Sleep disorders include not only common conditions such as insomnia and sleep apnea but also multidimensional issues, including circadian rhythm disruption and impaired sleep quality; the complex relationship between sleep disorders and BC requires further investigation [6, 7].

While numerous studies have explored the association between sleep and BC, findings remain inconsistent. Some observational studies have suggested that short or long sleep duration, as well as poor sleep quality, is associated with an increased risk of BC [8–10], whereas others have found no significant association [11, 12]. Additionally, sleep disorders may be closely linked to BC molecular subtypes, treatment stages, and patients’ psychological status [13–15]. These inconsistencies may stem from differences in research methods, population heterogeneity, and sleep assessment tools, highlighting the need for more rigorous studies.

The estrogen receptor (ER) plays a central role in the biology and clinical decision-making of BC [16]. Estrogen receptor-positive (ER+) BC is typically sensitive to endocrine therapy, with a relatively favorable prognosis [17], whereas estrogen receptor-negative (ER−) BC often has a poor prognosis and lacks endocrine therapy options [18]. In clinical practice, progesterone receptor (PR) and human epidermal growth factor receptor 2 (HER2) are usually evaluated alongside ER to classify major clinical phenotypes (Luminal A, Luminal B, HER2-positive, and triple-negative breast cancer (TNBC)) [19]. Among these, TNBC exhibits poorer biological features and prognosis due to the absence of effective targeted or endocrine therapies [20]. Using ER subtypes as the primary stratification not only offers direct clinical feasibility but also helps balance sample size and methodological consistency [21], thereby facilitating the exploration of heterogeneity in sleep-related effects and potential mechanisms.

To clarify the causal relationship between sleep disorders and BC, this study integrated cross-sectional data from NHANES and adopted MR analysis. Using large-scale population data and genetic instrumental variables, the study aimed to verify whether sleep disorders exert a causal effect on BC risk and further explore their differential effects on ER+ and ER− subtypes [22], providing scientific evidence for the clinical significance of sleep disorders in BC prevention and management.

## Materials and Methods

### Data source

This study integrated cross-sectional data from six cycles of NHANES 2007–2018 (2007–2008, 2009–2010, 2011–2012, 2013–2014, 2015–2016, and 2017–2018). The survey was conducted after obtaining approval from the Institutional Review Board of the National Center for Health Statistics (NCHS) and included questionnaires, physical examinations, and laboratory tests. All participants provided written informed consent. NHANES releases data every two years to ensure timeliness.

### Ethical considerations

This study used de-identified, publicly available NHANES data and summary-level genome-wide association study (GWAS) data. . The present secondary analysis did not involve direct participant contact or the collection of new human specimens.

### Cross-sectional study of NHANES

**Figure 1:**
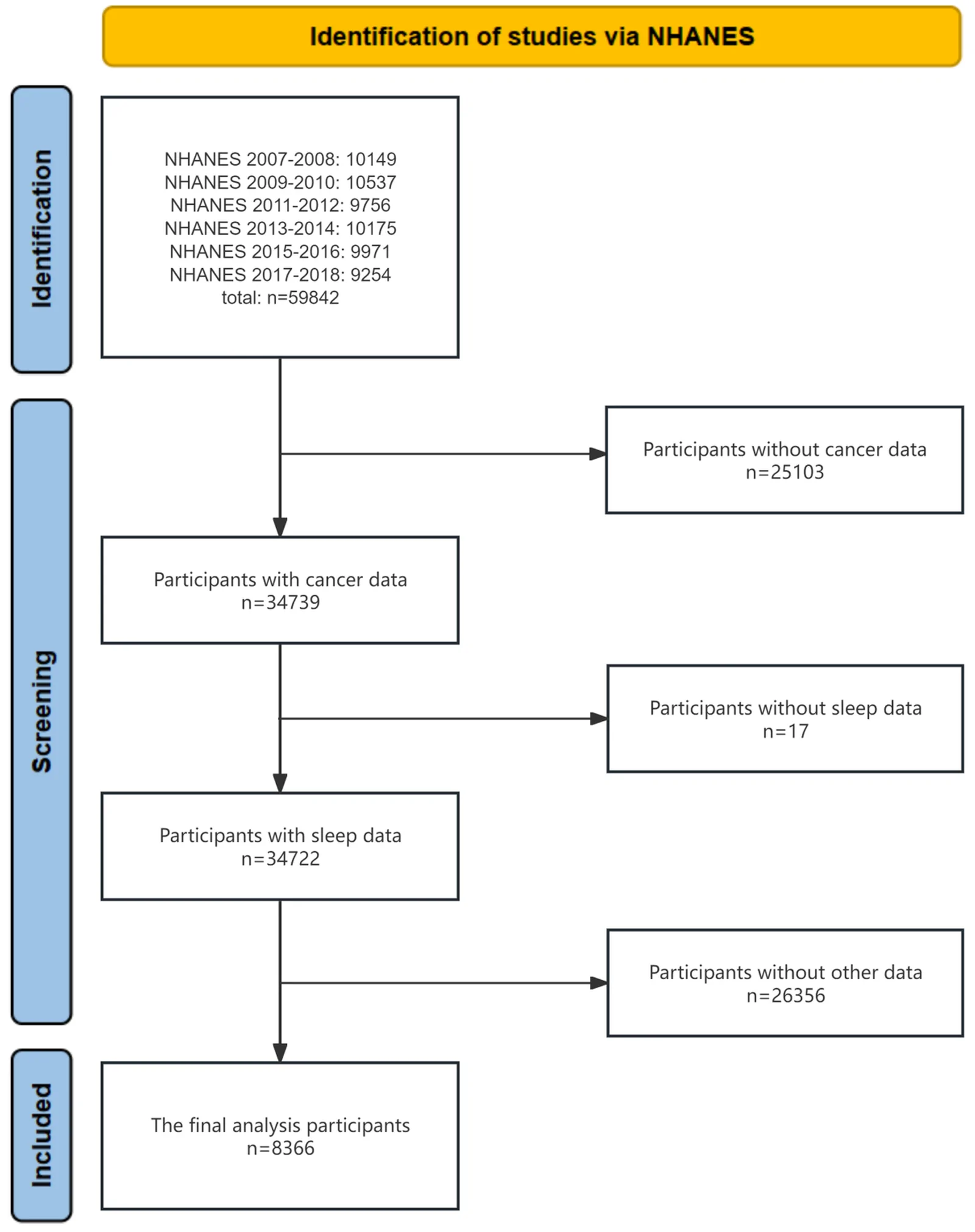
Flowchart of participants included in analyses.

This study used cross-sectional data from six cycles of NHANES 2007–2018 to explore the association between sleep disorders and BC. Sleep disorders were defined using the standardized question: “Have you ever been told by a doctor that you had trouble sleeping?” A response of “YES” was defined as the presence of sleep disorders, and “NO” as the absence. In the present study, this item was not validated against a clinical diagnosis or objective sleep measure and could encompass heterogeneous conditions, including insomnia symptoms, sleep-disordered breathing, transient sleep difficulty, and circadian disruption; it should therefore be interpreted as self-reported doctor-identified trouble sleeping rather than a diagnosis of a specific sleep disorder. BC was identified in two steps: first, participants were divided into the cancer group and non-cancer group based on their responses to the medical question: “Have you ever been told by a doctor or other health professional that you had cancer or a malignancy of any kind?”; subsequently, within the cancer group, participants were further classified into the BC group and non-BC group based on their answers to the question: “What kind of cancer?” BMI was obtained via standardized physical measurements, calculated as weight (kg) divided by height squared (m^2^). Covariates included age, gender, race (Mexican American, non-Hispanic Black, non-Hispanic White, other Hispanic, other race), educational level (less than 9th grade, 9–11th grade, high school graduate, some college courses or associate degree, college graduate and above), smoking status (participants who had smoked 100 cigarettes in their lifetime were considered smokers), drinking status (participants who had consumed ≥ 12 alcoholic beverages in their lifetime were considered drinkers), and status of hypertension and diabetes. The participant selection process is shown in Figure 1: the initial sample included 59,842 participants; after excluding males, samples not from 2007–2018, and those without cancer data, 34,739 participants remained; 17 participants without sleep data were further excluded, leaving 34,722 participants; finally, 26,356 participants without other data were excluded, resulting in a final sample of 8,366 participants for analysis. Exclusion criteria were: (1) males; (2) lack of relevant examination data; (3) patients with malignant tumors other than BC.

### Mendelian randomization (MR) analysis

To verify the causal relationship between sleep disorders and BC risk, two-sample MR analysis was employed. Exposure data were derived from summary data of a GWAS on sleep disorders in the European population (data set ID: finn-b-F5_SLEEP), including a total of 216,454 samples. Outcome data were obtained from two BC GWAS studies on European female populations: the ER+ BC data set (ID: ieu-a-1127) with a total sample size of 175,475 (69,501 cases and 105,974 controls); and the ER− BC data set (ID: ieu-a-1128) with a total sample size of 127,442 (21,468 cases (ncase) and 105,974 controls (ncontrol)). The selection of instrumental variables was based on three key assumptions: (1) genetic variations are strongly associated with the exposure factor (sleep disorders); (2) genetic variations are not confounded by confounders; (3) genetic variations affect BC outcomes only through sleep disorders [23, 24].

First, to assess the potential causal effect of sleep disorders on BC, single nucleotide polymorphisms (SNPs) significantly associated with sleep disorders were used as instrumental variables. Independent SNPs were selected from the whole genome with a significance threshold set at P < 5 × 10^−6^ [25]. Subsequently, based on reference data from the 1000 Genomes Project (European population), SNPs in linkage disequilibrium were excluded using the LDlinkR tool (criteria: r^2^ < 0.001 and distance > 10,000 kb) [26]. Further, pleiotropic SNPs associated with known confounders or directly linked to BC were screened and excluded using the PhenoScanner database [27].

After LD clumping and PhenoScanner screening, the exposure and outcome datasets were harmonized to align the effect alleles, and SNPs with incompatible alleles or ambiguous palindromic variants were excluded. Instrument strength was assessed using the F statistic (F = β^2^/SE^2^), and all retained SNPs had F statistics > 10, indicating a low likelihood of weak-instrument bias. Finally, four independent SNPs were retained for the MR analysis.

The IVW method was primarily used to estimate the causal effect of sleep disorders on BC [28]. To enhance the robustness of results, four supplementary methods were simultaneously applied: MR-Egger regression (for identifying and correcting pleiotropic bias), weighted median method (allowing up to 50% of instrumental variables to be invalid), weighted mode method, and simple mode method [29, 30]. Horizontal pleiotropy was evaluated using the MR-Egger intercept test (P > 0.05 indicated no significant pleiotropy), and heterogeneity was analyzed using Cochran’s Q test (P > 0.05 indicated no heterogeneity). In addition, leave-one-out sensitivity analysis (re-estimating the effect after sequentially excluding each SNP) and funnel plot drawing (assessing the symmetry of the results) were conducted to further ensure the reliability of causal inference [31].

### Statistical analysis methods

In this study, all continuous variables were described as weighted mean ± standard error, and categorical variables were expressed as frequency and weighted percentage. First, descriptive analysis was conducted on the baseline characteristics of the included participants. Subsequently, a weighted multivariable logistic regression model was used to analyze the association between sleep disorders and BC, with covariate-adjusted models constructed in three steps: model 1 (no variables adjusted); model 2 (adjusted for age, gender, race, and educational level); and model 3 (further adjusted for smoking, drinking, BMI, hypertension, and diabetes). For the weighted regression analyses, the complex multistage survey design of NHANES was accounted for using the 12-year combined MEC examination weight (WTMEC2YR/6), with SDMVSTRA and SDMVPSU specifying the strata and primary sampling units, respectively. To explore differences in the association between sleep disorders and BC across subgroups, additional subgroup analyses were conducted by age, race, BMI, educational level, drinking, smoking, hypertension, and diabetes status. All statistical analyses were performed using R software (version 4.4.3), with a significance level set at P < 0.05.

## Results

### NHANES cross-sectional analysis

A total of 8,366 participants were included in this study, among whom 290 had BC (Table 1). Compared with the non-cancer group, the BC group had a higher mean age, a higher proportion of non-Hispanic Whites, and higher prevalence of hypertension and diabetes. In terms of lifestyle factors, no significant differences were observed between the two groups in drinking, BMI, or folic acid level, but the BC group had a higher smoking rate. Among the BC group, 33.8% reported sleep disorders, compared with 27.9% in the non-cancer group (P = 0.035), suggesting a significant association between sleep disorders and BC.

**Table 1:** Comparison of Baseline Characteristics in Groups With and Without Breast Cancer

Variable	Group	Overall	Breast cancer	No cancer	P-value
N		8,366	290	8,076	
Gender (%)	Female	8,366 (100.0)	290 (100.0)	8,076 (100.0)	
Age (mean (SD))		50.97 (17.66)	67.69 (10.81)	50.37 (17.56)	< 0.001
Race (%)					< 0.001
	Mexican American	1,439 (17.2)	33 (11.4)	1,406 (17.4)	
	Non-Hispanic Black	2,002 (23.9)	51 (17.6)	1,951 (24.2)	
	Non-Hispanic White	2,633 (31.5)	150 (51.7)	2,483 (30.7)	
	Other Hispanic	1,053 (12.6)	26 (9.0)	1,027 (12.7)	
	Other Race including multi-racial	1,239 (14.8)	30 (10.3)	1,209 (15.0)	
Education (%)					0.222
	9–11th grade	1,110 (13.3)	35 (12.1)	1,075 (13.3)	
	College graduate or above	1,794 (21.4)	76 (26.2)	1,718 (21.3)	
	High school graduate	1,865 (22.3)	65 (22.4)	1,800 (22.3)	
	Less than 9th grade	1,060 (12.7)	28 (9.7)	1,032 (12.8)	
	Some college or AA degree	2,537 (30.3)	86 (29.7)	2,451 (30.3)	
Alcohol (%)					0.84
	No	3,351 (40.1)	114 (39.3)	3,237 (40.1)	
	Yes	5,015 (59.9)	176 (60.7)	4,839 (59.9)	
BMI (mean (SD))		30.24 (7.88)	29.87 (6.85)	30.26 (7.92)	0.409
High blood pressure (%)					< 0.001
	No	5,100 (61.0)	110 (37.9)	4,990 (61.8)	
	Yes	3,266 (39.0)	180 (62.1)	3,086 (38.2)	
Diabetes (%)					< 0.001
	Borderline	228 (2.7)	11 (3.8)	217 (2.7)	
	No	6,888 (82.3)	204 (70.3)	6,684 (82.8)	
	Yes	1,250 (14.9)	75 (25.9)	1,175 (14.5)	
Folic (mean (SD))		148.61 (145.69)	143.41 (133.88)	148.80 (146.10)	0.536
Smoke (%)					0.02
	No	6,162 (73.7)	196 (67.6)	5,966 (73.9)	
	Yes	2,204 (26.3)	94 (32.4)	2,110 (26.1)	
Sleep disorders (%)					0.035
	No	6,011 (71.9)	192 (66.2)	5,819 (72.1)	
	Yes	2,355 (28.1)	98 (33.8)	2,257 (27.9)	

BMI: body mass index; SD: standard deviation.

Three weighted logistic regression models were constructed to assess the association between sleep disorders and BC (Table 2). In model 1 (no covariates adjusted), participants with sleep disorders showed no significant increase in BC risk compared with those without sleep disorders (OR = 1.096, 95% CI: 0.775–1.551, P = 0.604). After adjusting for age, gender, race, and educational level in model 2, the association between sleep disorders and BC risk remained non-significant (OR = 0.859, 95% CI: 0.592–1.247, P = 0.426). In model 3, with additional adjustment for smoking, drinking, BMI, hypertension, and diabetes based on model 2, no significant statistical association was still observed between sleep disorders and BC risk (OR = 0.814, 95% CI: 0.553–1.196, P = 0.298).

**Table 2:** Association of Sleep Disorders and Breast Cancer

	Model 1		Model 2		Model 3	
	OR (95% CI)	P	OR (95% CI)	P	OR (95% CI)	P
No sleep disorders	Reference		Reference		Reference	
Sleep disorders	1.096 (0.775, 1.551)	0.604	0.859 (0.592, 1.247)	0.426	0.814 (0.553, 1.196)	0.298

OR: odds ratio; 95% CI: 95% confidence interval.

**Figure 2:**
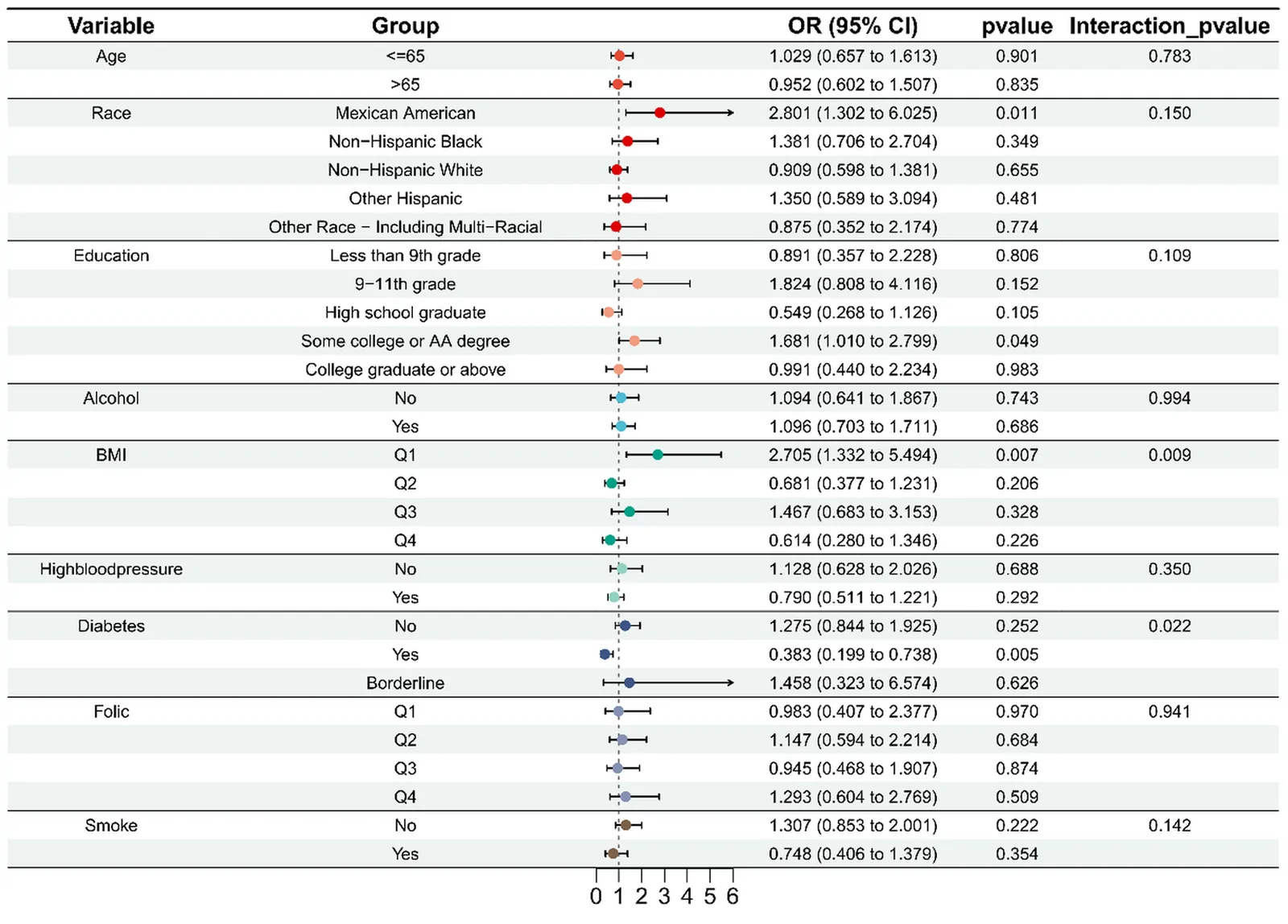
Forest plot of subgroup analysis. BMI: body mass index; OR: odds ratio; 95% CI: 95% confidence interval.

In the subgroup analysis of the association between sleep disorders and BC (Fig. 2), most variables showed no significant association. A significant positive correlation was observed in the Mexican American subgroup (OR = 2.801, 95% CI: 1.302–6.025). The lowest BMI quartile group (Q1) also showed a significant positive correlation (OR = 2.705, 95% CI: 1.332–5.494), with a significant interaction for BMI (P = 0.009). In diabetic patients, a significant negative correlation was found between sleep disorders and BC risk (OR = 0.383, 95% CI: 0.199–0.738), with a significant interaction (P = 0.022). No significant associations were observed in other subgroups, such as those stratified by age, education, drinking, or smoking.

### MR analysis

Five MR methods were used to evaluate the causal association between sleep disorders and ER+ BC (Table 3). The results showed that sleep disorders had no significant causal effect on ER+ BC risk, and the results of all methods were not statistically significant. For ER− BC, the IVW estimate indicated a modest positive association (OR = 1.070, 95% CI 1.013–1.130; P = 0.016), whereas the MR-Egger, weighted median, simple mode, and weighted mode estimates were not statistically significant (Table 4).

**Table 3:** MR Results of Sleep Disorders and ER+ Breast Cancer

ID.exposure	ID.outcome	Exposure	Outcome	MR method	OR (95% CI)	P-value
finn-b-F5_SLEEP	ieu-a-1127	Sleep disorders	ER+ breast cancer	MR-Egger	0.98(0.93, 1.03)	0.568
finn-b-F5_SLEEP	ieu-a-1127	Sleep disorders	ER+ breast cancer	Weighted median	0.98(0.94, 1.02)	0.349
finn-b-F5_SLEEP	ieu-a-1127	Sleep disorders	ER+ breast cancer	Inverse variance weighted	0.98(0.94, 1.02)	0.304
finn-b-F5_SLEEP	ieu-a-1127	Sleep disorders	ER+ breast cancer	Simple mode	0.98(0.93, 1.03)	0.537
finn-b-F5_SLEEP	ieu-a-1127	Sleep disorders	ER+ breast cancer	Weighted mode	0.98(0.94, 1.02)	0.465

ER+: estrogen receptor-positive; MR: Mendelian randomization; OR: odds ratio; 95% CI: 95% confidence interval.

**Table 4:** MR Results of Sleep Disorders and ER− Breast Cancer

ID.exposure	ID.outcome	Exposure	Outcome	MR method	OR (95% CI)	P-value
finn-b-F5_SLEEP	ieu-a-1128	Sleep disorders	ER− breast cancer	MR Egger	1.055 (0.978, 1.139)	0.301
finn-b-F5_SLEEP	ieu-a-1128	Sleep disorders	ER− breast cancer	Weighted median	1.067 (0.997, 1.141)	0.061
finn-b-F5_SLEEP	ieu-a-1128	Sleep disorders	ER− breast cancer	Inverse variance weighted	1.070 (1.013, 1.130)	0.016
finn-b-F5_SLEEP	ieu-a-1128	Sleep disorders	ER− breast cancer	Simple mode	1.061 (0.963, 1.168)	0.319
finn-b-F5_SLEEP	ieu-a-1128	Sleep disorders	ER− breast cancer	Weighted mode	1.060 (0.986, 1.139)	0.213

ER−: estrogen receptor-negative; MR: Mendelian randomization; OR: odds ratio; 95% CI: 95% confidence interval.

Table 5 presents the results of heterogeneity testing for the association between genetic variations of sleep disorders and ER− BC in GWAS data. For the MR-Egger method, the Q value was 0.231, the degrees of freedom (Q_df) were 2, and the corresponding P value (Q_pval) was 0.891; for the IVW method, the Q value was 0.489, the degrees of freedom were 3, and the P value was 0.921. The P values of both methods were much greater than 0.05, indicating that no significant heterogeneity was detected in this study, which supports the consistency and reliability of the results.

**Table 5:** Heterogeneity Testing of Sleep Disorders Genetic Variations in ER− Breast Cancer in a GWAS Dataset

ID.exposure	ID.outcome	Exposure	Outcome	Method	Q	Q_df	Q_pval
finn-b-F5_SLEEP	ieu-a-1128	Sleep disorders	ER− breast cancer	MR Egger	0.231	2	0.891
finn-b-F5_SLEEP	ieu-a-1128	Sleep disorders	ER− breast cancer	Inverse variance weighted	0.489	3	0.921

ER−: estrogen receptor-negative; GWAS: genome-wide association study; MR: Mendelian randomization.

The MR-Egger intercept analysis showed no evidence of directional horizontal pleiotropy (intercept = 0.006, SE = 0.012, P = 0.662; Table 6).

**Table 6:** Pleiotropy of Sleep Disorders Genetic Variations in ER− Breast Cancer in a GWAS Dataset

ID.exposure	ID.outcome	Exposure	Outcome	Egger_intercept	SE	P-value
finn-b-F5_SLEEP	ieu-a-1128	Sleep disorders	ER− breast cancer	0.006	0.012	0.662

ER−: estrogen receptor-negative; GWAS: genome-wide association study; SE: standard error.

**Figure 3:**
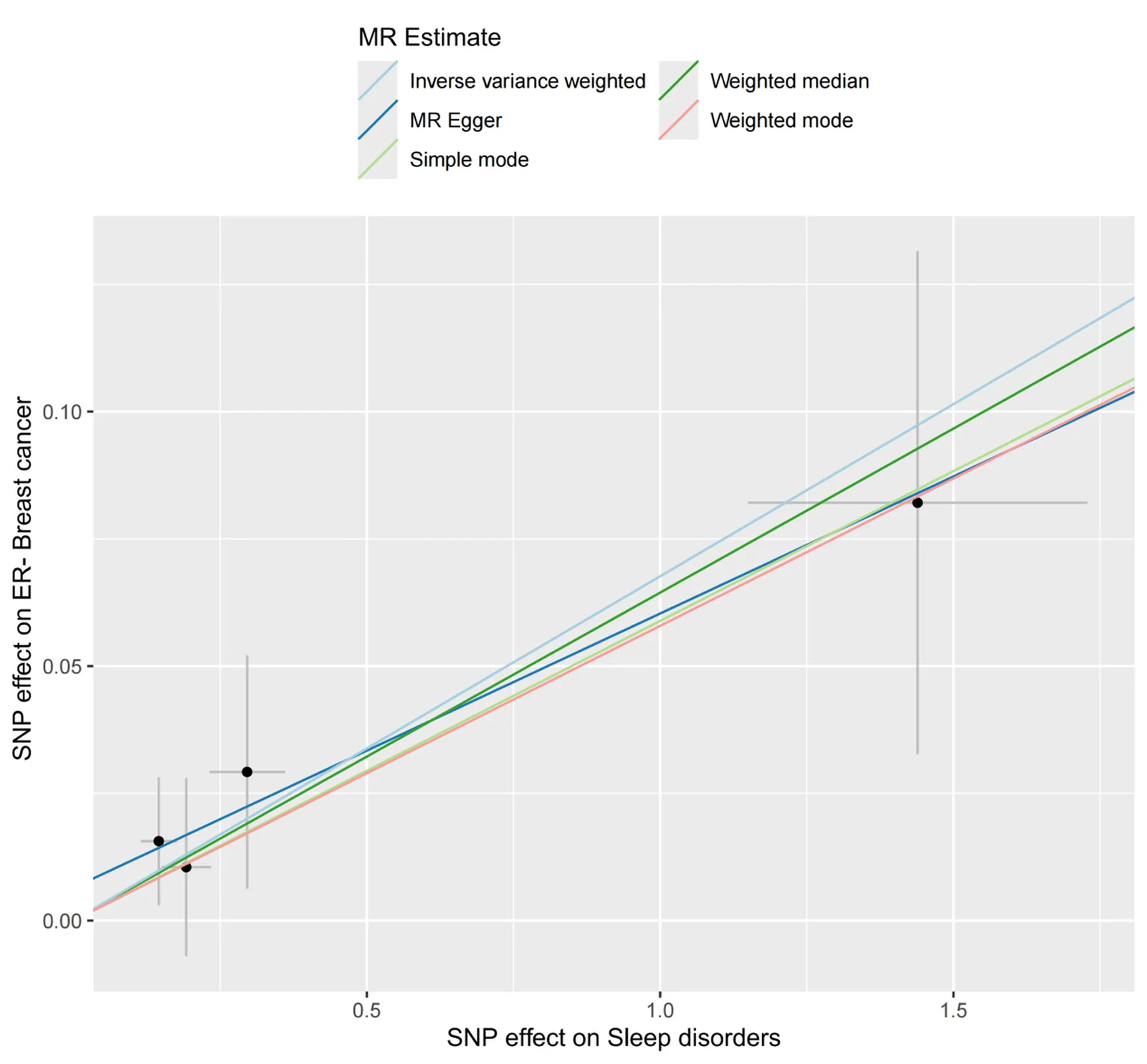
Scatter plot of sleep disorders and ER− breast cancer. The slope of each line denotes the estimated effect of per Mendelian randomization method. ER−: estrogen receptor-negative; MR: Mendelian randomization; SNP: single nucleotide polymorphism.

Five MR methods (IVW, MR-Egger, weighted median, weighted mode, and simple mode) were used to evaluate the causal relationship between sleep disorders and ER− BC. The scatter plot (Fig. 3) shows the association effects of SNPs with the exposure factor (sleep disorders) and outcome (BC). As shown in the figure, the slopes of all five MR methods were greater than zero, suggesting that sleep disorders may increase the risk of BC; the consistent positive slopes across methods further support the robustness of this positive association.

**Figure 4:**
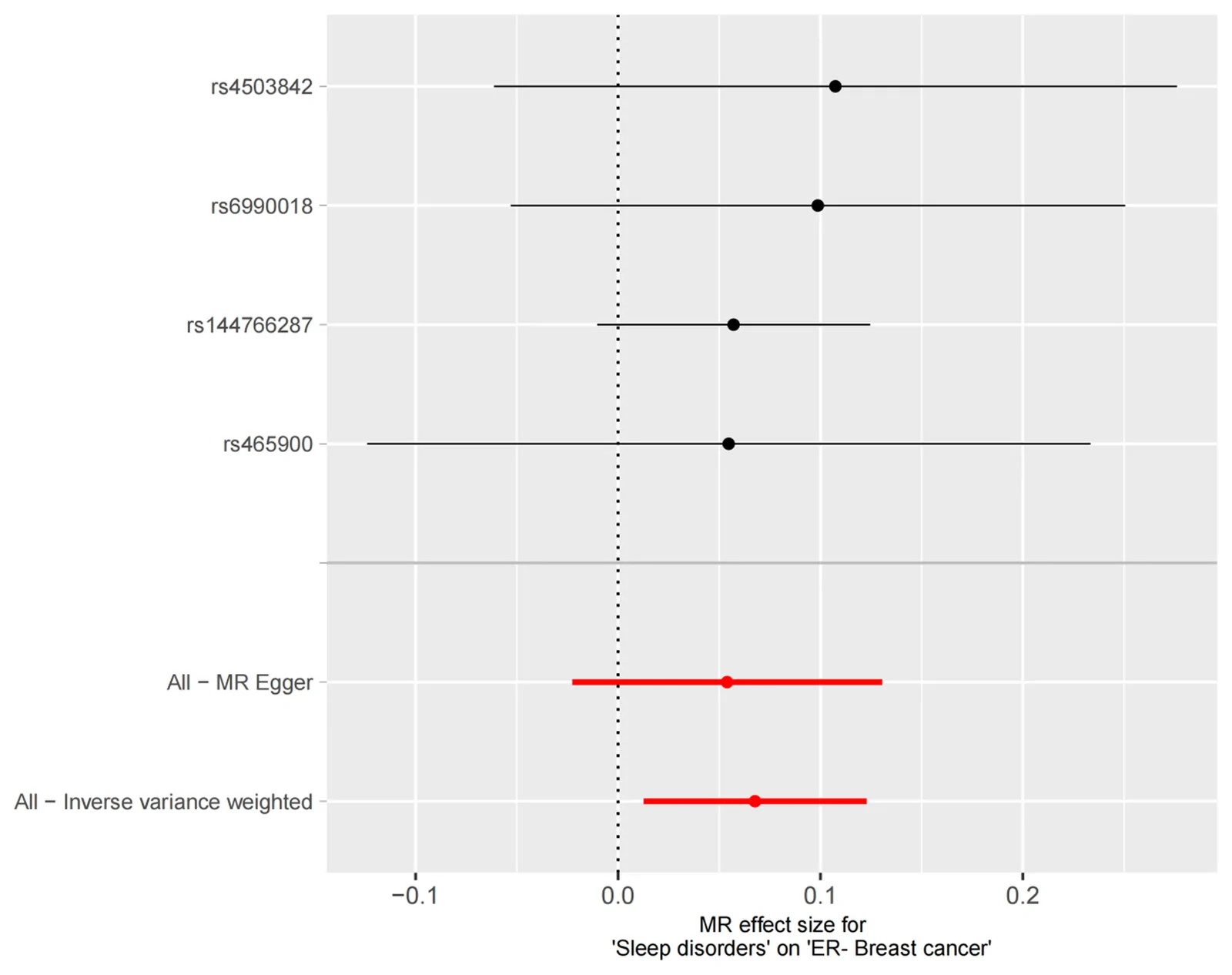
Forest plot of sleep disorder-related SNPs associated with ER− breast cancer. The red points demonstrate the integrated estimates using all SNPs together, using IVW method. Horizontal lines represent 95% confidence intervals. ER−: estrogen receptor-negative; IVW: inverse variance weighted; MR: Mendelian randomization; SNP: single nucleotide polymorphism.

Figure 4 shows the forest plot of MR analysis results of sleep disorder-related SNPs on ER− BC. The plot includes effect size estimates for multiple SNPs and two integrated analysis methods (MR-Egger and IVW). The forest plot shows that the result of the IVW method based on all SNPs was greater than zero, suggesting that sleep disorders may be a risk factor for ER- BC.

**Figure 5:**
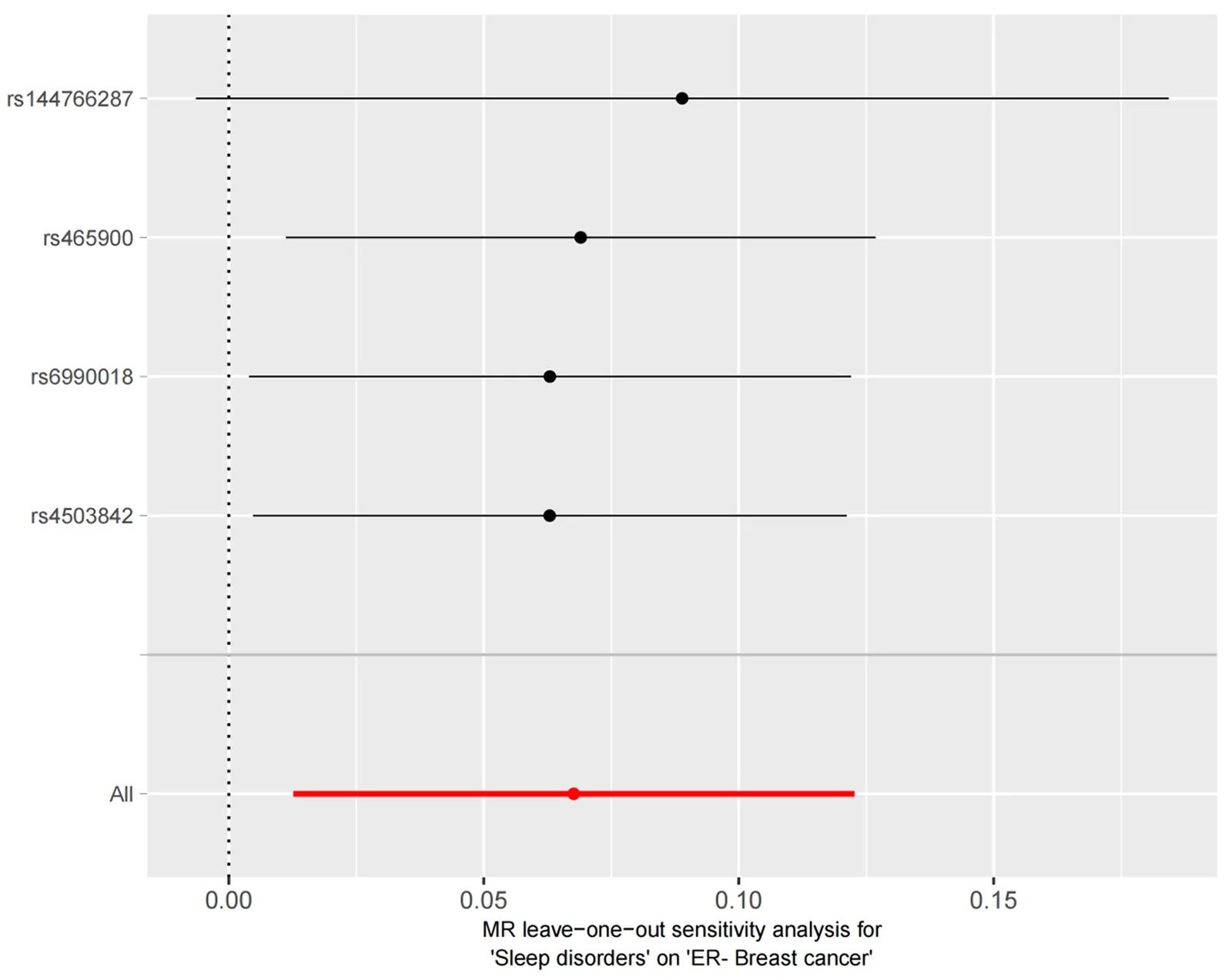
Leave-one-out analysis of the causal association of sleep disorders and ER− breast cancer. Black points depict the IVW method was used to assess the causal effect, excluding single specific variant from the analysis. The red point denotes the inverse-variance weighted estimate using all SNPs. ER−: estrogen receptor-negative; IVW: inverse variance weighted; MR: Mendelian randomization.

The results of Figure 5 (leave-one-out sensitivity analysis) showed that the effect estimates obtained after sequentially excluding each SNP had minimal differences compared with the combined effect size obtained by IVW using all SNPs, indicating that the results of this study were robust and no single SNP exerted an excessive impact on the overall MR analysis conclusion.

**Figure 6:**
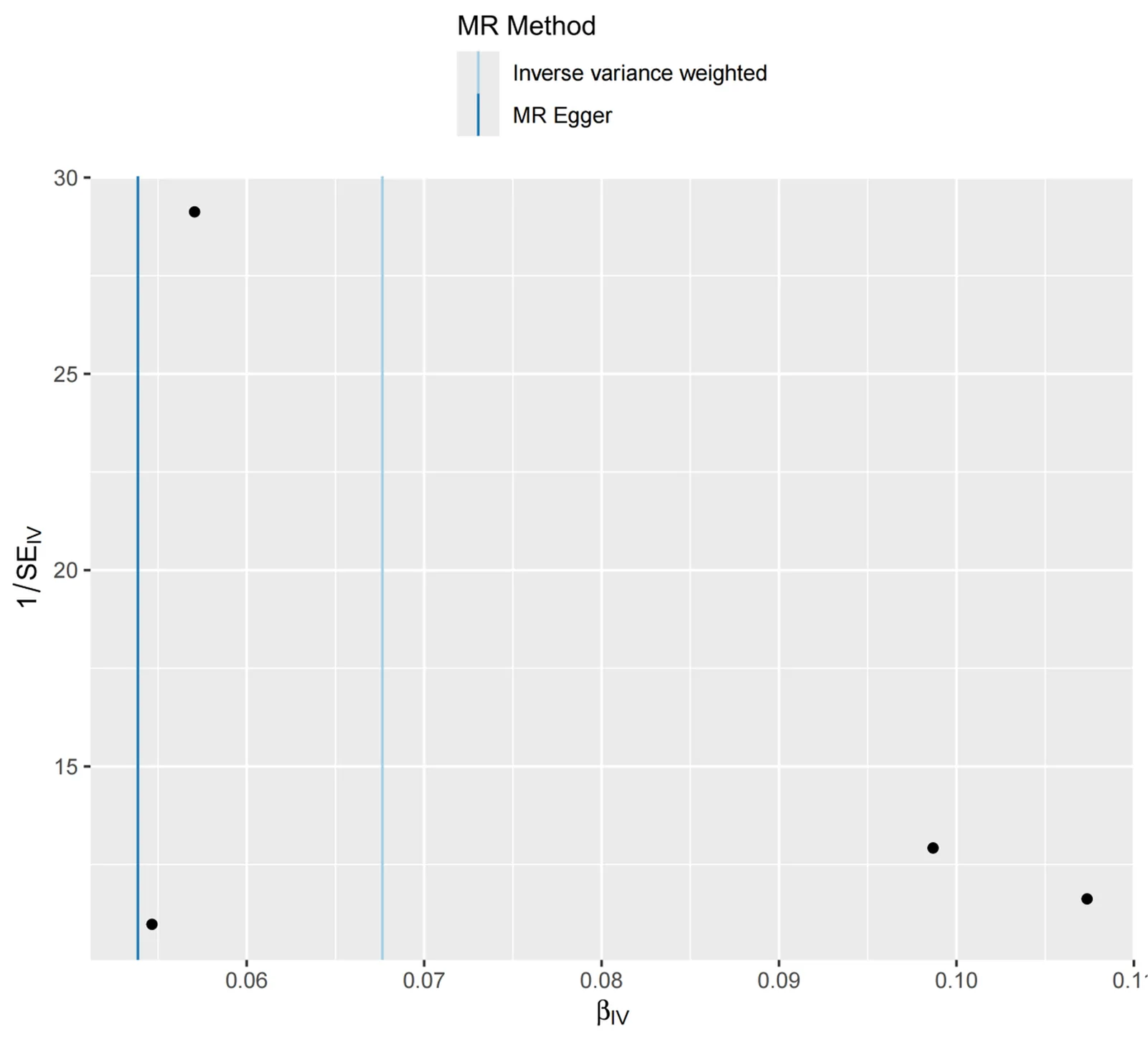
Funnel plot. Vertical lines represent estimates with all SNPs. Symmetry in the funnel plot demonstrates no obvious horizontal pleiotropy. MR: Mendelian randomization; SNP: single nucleotide polymorphism.

The effect estimates of all SNPs in the funnel plot were symmetrically distributed on both sides of the IVW effect line, indicating no heterogeneity (Fig. 6).

## Discussion

By integrating large-scale cross-sectional survey data and MR analysis, this study systematically explored the complex relationship between sleep disorders and BC risk. The core findings of the study included three aspects: first, in the NHANES cross-sectional data, although the proportion of BC patients reporting sleep disorders was higher, no significant statistical association was found between sleep disorders and overall BC risk after adjusting for confounders. Second, subgroup analysis revealed significant population heterogeneity, with the association between sleep disorders and BC varying across subgroups defined by race, BMI, and diabetes status. Most importantly, MR analysis provided causal evidence at the genetic level, confirming that sleep disorders are a specific risk factor for ER− BC but have no causal association with ER+ BC.

The different NHANES and MR findings are not necessarily contradictory. NHANES assessed prevalent BC and a single self-reported sleep item at one time point, so exposure misclassification, reverse causation, residual confounding, selection or survivor bias, and limited power could attenuate or distort the observed association. MR instead estimates the association of lifelong genetic liability in a European-ancestry population and is less vulnerable to some forms of confounding but depends on valid instruments. Moreover, NHANES evaluated BC overall, whereas the MR signal was limited to ER− BC and could therefore be diluted in the overall-BC analysis.

Exploratory subgroup analyses suggested possible heterogeneity by race, BMI, and diabetes status. However, the small number of BC cases within subgroups, multiple uncorrected comparisons, and possible residual confounding limit the stability of these estimates. These findings should therefore be considered hypothesis-generating.

Previous studies have documented a high prevalence of sleep disturbances among BC survivors and described bidirectional mechanisms through which cancer may disrupt sleep [32, 33]. Population studies have also examined sleep duration, BMI, and cancer risk, although prospective evidence for BC remains inconsistent [34, 35]. Racial and ethnic differences in sleep and circadian responses have been reported [36], and experimental evidence suggests that sleep loss can alter the DNA binding of core circadian-clock proteins [37]. The diabetes subgroup finding should be interpreted cautiously because insulin resistance is associated with BC risk and outcomes [38, 39], whereas metformin has been investigated for potential antineoplastic effects [40–42]. Differences in healthcare contact and cancer detection among patients with diabetes may also influence observed associations [43]. These reports provide biological and epidemiological context only and do not validate the exploratory subgroup associations observed in the present study.

Employing MR approach, the causal effects of sleep disorders on different ER BC subtypes were explored at the genetic level. The results showed that sleep disorders exerted a significant positive causal effect on ER− BC but no significant effect on ER+ BC. This finding of a causal association between sleep disorders and ER− BC may provide a new entry point for etiological exploration and risk prevention and control research of this subtype, as ER− BC is typically more aggressive and has limited treatment options [44]. Previous MR studies have also suggested that sleep characteristics (e.g., sleep duration, chronotype) have a causal association with BC risk, but most did not stratify by molecular subtype [45, 46]. This study clearly indicates, via genetic instrumental variables, that sleep disorders may specifically promote the development of ER− BC.

MR analysis identified a positive causal association between sleep disorders and ER− BC risk, but its biological mechanism remains unclear. Multiple studies have suggested that sleep disorders may affect the onset and progression of BC through various pathways, particularly in the ER− subtype. First, sleep disorders can lead to reduced melatonin secretion, thereby losing its inhibitory effect on the estrogen signaling pathway [47, 48]; this may particularly affect ER+ tumors, whereas ER− tumors may be more affected through non-hormonal pathways [49, 50]. Second, intermittent hypoxia caused by sleep-disordered breathing can activate the hypoxia-inducible factor 1-alpha (HIF-1α) pathway, promoting angiogenesis and tumor progression, and is associated with aggressive tumor characteristics [51–54]. Genetic factors may also modulate the association between sleep and cancer risk [55, 56]. Treatment-related factors (e.g., chemotherapy or endocrine therapy) may further disrupt the sleep–wake cycle and melatonin secretion [57, 58], while psychological factors (e.g., depression, anxiety) may exacerbate the vicious cycle between sleep disorders and cancer via neuroendocrine pathways [59–61]. Currently, these mechanisms are mostly explored in isolation; the interactions between these mechanisms in ER− BC remain unclear, and there is a lack of subtype-specific mechanism validation. Future research should focus on the biological characteristics of ER− BC to explore the synergistic or regulatory relationships between these mechanisms, providing more precise theoretical support for the development of ER− BC prevention or intervention strategies targeting sleep-related pathways.

Notably, no causal effect of sleep disorders was observed in ER+ BC in this study, which may be related to the dominant role of the estrogen pathway in ER+ tumors; sleep-related mechanisms may affect tumor development primarily through non-hormonal pathways [62]. Additionally, the association between sleep disorders and BC may exhibit a complex pattern due to interactions between genetic background, environmental exposure, and behavioral factors [7]; future research should combine multi-omics data and longitudinal design for further validation.

A strength of this study is the complementary use of population-based and MR analyses to examine the sleep–BC association from different perspectives. Nevertheless, several limitations should be acknowledged. The NHANES exposure was a single self-reported item that did not distinguish specific sleep disorders, and the cross-sectional design cannot establish whether sleep problems preceded BC, allowing reverse causation. Exclusion of 51,476 of 59,842 participants (86.0%) for sex, survey cycle, outcome, exposure, or covariate availability may have introduced selection bias, while reliance on prevalent BC cases may have introduced survivor bias. Only 290 BC cases were included, limiting power and precision, particularly in subgroup analyses; residual confounding and multiple uncorrected comparisons further require the subgroup findings to be regarded as exploratory. The MR analysis was restricted to European-ancestry data and used only four instruments; the ER− estimate was modest and statistically significant only with IVW, so it should not be viewed as definitive proof of causality. Future studies should use prospective designs, validated and objective sleep measures, and larger subtype-specific samples.

### Conclusion

This study, integrating NHANES 2007–2018 cross-sectional data and two-sample MR analysis, found no overall significant association between sleep disorders and BC risk after covariate adjustment (OR = 0.814, 95% CI: 0.553–1.196, P = 0.298), despite a higher prevalence of sleep disorders among BC patients (33.8% vs. 27.9%, P = 0.035). Subgroup analyses revealed marked heterogeneity, with positive associations in Mexican Americans and low-BMI individuals, and a negative association in diabetic patients. MR, however, provided causal evidence of a subtype-specific effect: sleep disorders significantly increased the risk of ER− BC (IVW OR = 1.070, 95% CI: 1.013–1.130, P = 0.016), while showing no causal relationship with ER+ disease (IVW OR = 0.98, 95% CI: 0.94–1.02, P = 0.304). These findings, supported by robust sensitivity analyses, suggest that sleep disorders may selectively contribute to the etiology of ER− BC and warrant consideration in subtype-targeted prevention strategies.

## Data Availability

The authors declare that data supporting the findings of this study are available within the article.

## Abbreviations

BC: breast cancer
BCAC: Breast Cancer Association Consortium
BMI: body mass index
CI: confidence interval
ER: estrogen receptor
ER+: estrogen receptor-positive
ER−: estrogen receptor-negative
GWAS: genome-wide association study
HER2: human epidermal growth factor receptor 2
HIF-1α: hypoxia-inducible factor 1-alpha
IRB: Institutional Review Board
IVW: inverse variance weighted
MR: Mendelian randomization
NCHS: National Center for Health Statistics
NHANES: National Health and Nutrition Examination Survey
OR: odds ratio
PR: progesterone receptor
SD: standard deviation
SNP: single nucleotide polymorphism
TNBC: triple-negative breast cancer
